# The Effects of Mindfulness-Based Intervention on Shooting Performance and Cognitive Functions in Archers

**DOI:** 10.3389/fpsyg.2021.661961

**Published:** 2021-06-24

**Authors:** Tsung-Yi Wu, Jui-Ti Nien, Garry Kuan, Chih-Han Wu, Yi-Chieh Chang, Hsueh-Chih Chen, Yu-Kai Chang

**Affiliations:** ^1^Department of Combat Sport, National Taiwan University of Sport, Taichung, Taiwan; ^2^Graduate Institute of Athletics and Coaching Science, National Taiwan Sport University, Taoyuan, Taiwan; ^3^Exercise and Sports Science Programme, School of Health Sciences, Universiti Sains Malaysia, Kelantan, Malaysia; ^4^Physical Education Center, Chung Shan Medical University, Taichung, Taiwan; ^5^Department of Educational Psychology and Counseling, National Taiwan Normal University, Taipei, Taiwan; ^6^Institute for Research Excellence in Learning Science, National Taiwan Normal University, Taipei, Taiwan; ^7^Department of Physical Education, National Taiwan Normal University, Taipei, Taiwan

**Keywords:** mindfulness intervention, fine motor, cognitive functions, dose-response, archer

## Abstract

This study investigated the effects of a mindfulness-based intervention (MBI) called mindfulness-based peak performance (MBPP) on athletic performance and cognitive functions in archers, as well as the role of psychological status and the dose-response relationship of MBPP in archery performance. Twenty-three archers completed a simulated archery competition and the Stroop task prior to and after MBPP training, which consisted of eight sessions over four weeks, while the mindfulness and rumination levels of the archers were assessed at three time points, namely, before, at the mid-point of, and after the MBPP program. The results revealed that the MBPP program significantly improved the shooting performance (*p* = 0.002, *d* = 0.27), multiple cognitive functions (*ps* < 0.001, *d* = 0.51~0.71), and mindfulness levels of the archers on the post-test, compared to the pre-test (*p* = 0.032, *η*_*p*_^2^ = 0.15 for general; *p* = 0.004, *η*_*p*_^2^ = 0.22 for athletic). Additionally, negative ruminations level was decreased from the pre-test to the middle-test and post-test (*ps* < 0.001, *η*_*p*_^2^ = 0.43). These findings provide preliminary evidence to support the view that MBPP could serve as a promising form of training for fine motor sport performance, cognitive functions, and specific psychological status, such that it warrants further study.

## Introduction

Mental ability is one of primary foundations for successful sport performance (Kim et al., 2015), and mindfulness has recently been recognized as a novel aspect of sport-related mental ability (Jones et al., 2020). Mindfulness is believed to reflect both focus on and awareness of one's moment-to-moment experiences through a non-judgmental attitude (Kabat-Zinn, 2003; Creswell, 2017). Mindfulness also is described as a trait or disposition that involves the amount of tendency of the individual to be mindful in daily life (Birrer et al., 2012). Relatedly, mindfulness levels have been considered a key factor in ensuring the optimal psychological status necessary to achieve peak performance in fine motor sports (Gooding and Gardner, 2009; Zhang et al., 2016).

Mindfulness-based interventions (MBIs) involving mental concepts, specific exercises, and class discussions that are intended to induce positive effects in terms of individual focus, mindfulness-related body sensations, cognitive factors, and emotional regulation (Kabat-Zinn, 1982, 2003; Creswell et al., 2019), have been observed to efficiently cultivate mindfulness through regular practice in athletes (Birrer et al., 2012; Josefsson et al., 2019; Nien et al., 2020). Additionally, MBIs have been found to directly affect fine motor sport performances in sports involving precision, accuracy, and dexterity (Bühlmayer et al., 2017; Noetel et al., 2019), such as shooting (John et al., 2011) and dart throwing (Zhang et al., 2016), in addition to being linked to enhanced psychological parameters (Birrer et al., 2012; Bühlmayer et al., 2017), such as improved flow experiences, self-confidence, and competitive anxiety, in archers (Kaufman et al., 2009; Thompson et al., 2011).

The beneficial effects of MBIs on sport performance may be derived through improvement of cognitive functions (Scharfen and Memmert, 2019; Nien et al., 2020). A recent meta-analysis reported superior cognitive functions [e.g., executive function (EF), visuoperceptual function], with small to medium positive effect sizes, in experts and elite athletes compared with non-elite athletes (Scharfen and Memmert, 2019). Among the variety of cognitive functions, EF is a higher-order cognitive function responsible for top-down processes aimed at achieving goal-directed behaviors (Audiffren and André, 2019; Etnier and Chang, 2019; Chen et al., 2020). Previous studies have shown that there is a link between EF and peak performance in athletes (Sakamoto et al., 2018; Ishihara et al., 2019). EF contains three distinguishable components (e.g., inhibitory control, working memory and cognitive flexibility) (Diamond and Ling, 2016), and the inhibitory control aspect of EF may play a crucial role in fine motor sports (Jacobson and Matthaeus, 2014), because athletes competing in these sports must maintain extremely high levels of concentration on their targets while also ignoring internal and external distractions (Diamond and Ling, 2016). López-Navarro et al. (2020) found that a long-term mindfulness exercise integrated with rehabilitation treatment improved inhibitory control in patients with psychotic disorder. Nien et al. (2020) further reported that a five weeks MBI improved participant performance on inhibition-related conditions (i.e., the incongruent condition) of the Stroop task and increased the durations at which participants completed an exhaustion running test, suggesting associations among MBI, EF, and sport performance.

The beneficial effects of MBIs on sport performance may be associated with psychological processes (Birrer et al., 2012). Josefsson et al. (2017) suggested that less rumination is an essential mechanism between mindfulness and coping skills in a sports context. Rumination is regarded as a psychological process that repetitively and passively focuses on distress and is associated with a variety of maladaptive cognitive styles (e.g., depression and pessimism), and may connect to sport-related coping factors in athletes (e.g., concentration and arousal regulation) (Birrer et al., 2012). It should be noted, moreover, that rumination is associated with EF and that lower EF is associated with higher levels of negative rumination and with negative affect (De Lissnyder et al., 2010). However, few studies have simultaneously examined the influences of rumination and inhibitory control in the relationship between MBIs and fine motor sport performance.

Another research gap relates to the minimal doses of MBIs necessary to achieve the desired effects. There has been shown that a dose-response relationship obtains between the number of sessions per week of psychotherapy intervention and depression symptoms (i.e., an increase from one to two sessions per week increased the effect size) in the past meta-regression analysis (Cuijpers et al., 2013). In mindfulness research, Creswell (2017) suggests that MBIs lasting at least 8 weeks have greater effects on adverse negative psychological outcomes (e.g., anxiety). However, it should be noted that the majority of related studies have adopted the mindfulness-based stress reduction (MBSR, Kabat-Zinn, 1982, 2003) principle as a standard program, and MBSR entails the use of an 8 weeks course with one session per week lasting 2–2.5 h. Meanwhile, Roos et al. (2019) evaluated the effect of eight MBI sessions within 4 weeks on mental health in patients with substance use disorders and found that participants who attended more than two sessions could achieve significantly better mental health and higher mindfulness at discharge. A meta-regression study further suggested that mindfulness outcomes could be predicted by greater contact with, intensity of, and actual use of MBIs, but no significance in other psychological outcomes (Strohmaier, 2020). These studies suggest that an MBI course may be shorter than 8 weeks, as long as the minimum number of necessary sessions is included. However, whether a dose-response relationship exists between the number of sessions in MBIs and mindfulness levels, including account for rumination, remains unclear.

The present study thus sought to examine the effects of an MBI called mindfulness-based peak performance (MBPP) on archery performance while also examining the roles of mindfulness level, cognitive function (e.g., inhibitory control), and rumination, as well as the associated dose-response relationships in terms of the number of sessions in the MBPP program. We hypothesized that the MBPP program would enhance the shooting scores, Stroop task performance, and mindfulness levels of the participating archers, in addition to reducing their rumination levels. Additionally, the dose-response relationship between the MBPP program and behavioral and psychological outcomes was also investigated.

## Materials and Methods

### Participants

Twenty-three competitive archery athletes (19 men and 4 women, mean age = 20.64 ± 1.20 years) were recruited from National Taiwan University of Sport, and their mean experience in their sport was 7.47 ± 1.89 years. The participants were included in the study based on the following criteria: (a) no prior experiences of mindfulness related training (e.g., meditation, yoga, or Tai Chi); (b) no history of psychiatric or neurological disorders; (c) not taking medicines affecting the central nervous system or brain; and (d) normal or corrected-to-normal vision and no color blindness. Furthermore, the participants filled in a written informed consent form approved by the Center for Research Ethics of National Taiwan Normal University before the initiation of the experiment.

### Measurements

#### Shooting Performance

An Olympic archery individual competition was simulated to assess the shooting performance of the participants. The shooting position of the target was set according to international standards, with the distance from the archer to the target being 70 meters, and each archer shot a total of 72 arrows (in six ends, or groups, of 12 arrows). The score for each shot was determined by how close the arrow was to the center of the target. The participants were assessed at a pre-test and post-test (which followed the 8 sessions of the MBPP program), both of which occurred during the regular archery training season and took place on the outdoor archery training field of National Taiwan University of Sport. In order to reduce the disturbances caused by wind, a vane anemometer was used to measure the wind speed, and it was found that the wind speeds during the pre-test ranged from 0.1 to 1.2 m/s while those during the post-test ranged from 0.0 to 1.3 m/s. The final results of each shooting test were obtained from the coach after the simulated competition, with higher shooting scores indicating greater athletic performance.

#### EF: Stroop Task

The paper-pencil version of the Chinese Stroop color-word task was used to assess multiple aspects of cognitive functioning (i.e., information processing speed, selective attention, and inhibitory control) (Chu et al., 2016). The Stroop task consists of three types of conditions: (a) neutral (in which colored squares are presented); (b) congruent (in which the meaning of the words presented corresponds to the color of ink in which they are presented); and (c) incongruent (in which the meaning of the words presented does not correspond to the color of ink in which they are presented). Both of the latter conditions consist of a string of Chinese color-words [i.e., “紅” (red), “綠” (green), and “藍” (blue)] that are presented individually on A4 size paper (210 mm × 297 mm; 10 rows, 5 columns), and each condition includes 50 stimuli.

During the Stroop task, the participants were asked to ignore the meaning of each word and to verbally name the color of the ink in which each word was presented as rapidly and accurately as possible, going from top to bottom and left to right. If the participant being tested made a mistake, he or she was asked to try again until the correct color was named. The reaction times were recorded from the first stimulus to the final stimulus in each condition as the score indexing the cognitive performance.

#### Self-Reported Psychological Outcomes

##### Chinese Mindful Attention Awareness Scale

The general mindfulness level of each participant was measured using the Chinese version of the Mindful Attention Awareness Scale (CMAAS) (Chang et al., 2011), a revised version of the original Mindful Attention Awareness Scale (MAAS), which is a widely adopted mindfulness scale designed to assess mindfulness levels in general states (Brown and Ryan, 2003). The CMAAS is a 15-item questionnaire that uses a 6-point Likert scale ranging from 1 (almost never) to 6 (almost always) for each item. The items include statements such as “It seems I am ‘running on automatic' without much awareness of what I'm doing” and “I find myself doing things without paying attention.” Higher scores indicate greater general dispositional mindfulness. In this study, the CMAAS produced satisfactory internal consistency across the three time points (α = 0.82~0.93).

##### Chinese Mindfulness Inventory in Sport

The athletic mindfulness level of each participant was measured using the Chinese version of the Mindfulness Inventory in Sport (CMIS) (Peng and Shen, 2016), a revised version of the original Mindfulness Inventory in Sport (MIS) developed by Thienot et al. (2014). The CMIS consists of two sub-dimensions, attentional control and non-judgment, and is intended to assess mindfulness levels associated with sports contexts. The attentional control sub-dimension consists of ten items (e.g., “I pay attention to the type of emotions I am feeling”), while the non-judgment sub-dimension consists of five items (e.g., “When I become aware that I am really upset because I am losing, I criticize myself for reacting this way”). The CMIS thus has a total of 15 items, with a 6-point Likert scale ranging from 1 (rarely) to 6 (every time) used for each item. Higher scores indicate greater athletic mindfulness levels. In the present study, there was satisfactory internal consistency for the total CMIS (α = 0.83~0.94), the attentional control sub-dimension (α = 0.80~0.92), and the non-judgment sub-dimension (α = 0.76~0.86) across the three time points.

##### Chinese Multidimensional Rumination Questionnaire

The Chinese version of the Multidimensional Rumination Questionnaire (CMRQ) (Tu and Hsu, 2008), a revised version of the original Multidimensional Rumination Questionnaire (MRQ) developed by Fritz (1999), was utilized to assess each participant's level of rumination. The CMRQ is designed to measure three sub-dimensions, namely, emotional-focused rumination (13 items regarding thinking about thoughts, feelings, or affect in relation to negative experiences), meaning-searching rumination (7 items regarding searching for the meaning of negative experiences or events), and instrumental rumination (5 items regarding thinking about what approach can be used to address negative events), with a 5-point Likert scale ranging from 1 (almost never) to 5 (almost always) used for each item. There was satisfactory internal consistency for the emotional-focused rumination sub-dimension (α = 0.95~0.97), the meaning-searching rumination sub-dimension (α = 0.79~0.91), and the instrumental rumination sub-dimension (α = 0.90~0.91) across the three time points.

### Mindfulness-Based Peak Performance Program

The Mindfulness-Based Peak Performance (MBPP) program utilized in the current study was adopted from the mindfulness components of MBSR (Kabat-Zinn, 1982, 2003) (i.e., awareness, paying attention on purpose, being in the present moment, and experiencing things non-judgmentally), as well as our own previous MBI program (Nien et al., 2020). The MBPP program consisted of a total of eight 60-min sessions conducted twice per week over 4 weeks, the aim of which was to enhance mindfulness levels and performance. The sessions covered two fundamental concepts, namely, mindfulness in general and mindfulness in sports contexts, and included six core modules regarding various findings of brain science related to successful performance, such as findings about habits, stress, rest, attention, emotion, and executive functioning. Additionally, the MBPP sessions also focused on mindfulness exercises, which included mindful check-ins, the raisin exercise, mindful breathing, body scanning, seated meditation, mindful walking, mindful listening, and mindful Bagua Dǎo yin, with each exercise corresponding to the given session topic.

The MBPP course was conducted by a scholar with a Ph.D. in sport and exercise psychology and three certificated instructors with master's degrees in sport psychology. Each session of the MBPP program started with a brief story about performance aimed at inducing the learning motivation of the participants, after which the content of the preceding session and associated homework were reviewed and discussed, followed by a theoretical discussion introducing a new topic and then a mindfulness exercise. Finally, each session concluded with a discussion of the specific application of mindfulness concepts and skills during sports competition situations (e.g., mindful breathing before shooting or mindful walking on the way from a shooting position to the target) (Nien et al., 2020), as well as providing advice on the barriers suffered during the homework.

To enhance adherence (Zhang et al., 2016), the team coach would guide the athletes in practicing the mindfulness skills for 15-min in a group before regular training, and the athletes were also encouraged to perform the assigned 20-min daily home practice. Moreover, all of the athletes received an MBPP program handbook that included a scheduled timetable and notes regarding the mindfulness exercises. The MBPP program was conducted in a classroom of National Taiwan University of Sport. The Appendix I presents a summary outline of the MBPP program protocol.

### Procedure

A one-group pretest-posttest design was employed to examine the effects of the MBPP program on the shooting performance of the participating archers. An orientation was conducted to thoroughly explain the nature of the study so that both the coach and athletes would understand the experimental procedure. The first data collection (i.e., the pre-test) was conducted before the first MBPP program session, the second data collection (i.e., the middle-test) was conducted immediately after the fourth session, and the third data collection (i.e., the post-test) was conducted immediately after the eighth session. MBPP sessions started at 1:00 p.m., and all measurements at three time points were conducted between 3:00 p.m. and 5:00 p.m. Participants were also asked not to consume caffeine for at least 3 h before testing. In addition, so as to avoid the practice effect from tests (Chen et al., 2013), the measures of shooting performance and cognitive functions were only administered at the pre-test and post-test, the measures of psychological outcomes including the mindfulness and rumination levels were administered at the pre-test, middle-test, and post-test.

### Statistical Analysis

SPSS software version 26.0 was used in this study. Descriptive statistics were utilized to calculate the means and standard deviations of the demographic data. Normal distribution confirmation was used by applying the Shapiro-Wilk test, and all of the data were normally distributed across each of the time points. A paired sample *t*-test was conducted to compare the pre-test and post-test shooting score and cognitive function performances. The respective results of the three psychological outcome measures (i.e., the CMAAS, CMIS, and CMRQ) were compared using a one-way analysis of variance (ANOVA), with Bonferroni adjustments being performed for multiple comparisons in the further analysis, to determine whether the MBPP program affected the psychological outcomes over time. Cohen's *d* and partial eta-squared (*η*_*p*_^2^) are presented was used to estimates the effect size for *t*-test and one-way ANOVA, respectively. In addition, Pearson correlation analysis was performed for post-test to assess the relationships among the shooting score, cognitive function performances, and the three psychological outcomes. A *p*-value < 0.05 was set as the level of statistical significance for all analyses.

## Results

### Shooting Performance

**Figure 1:**
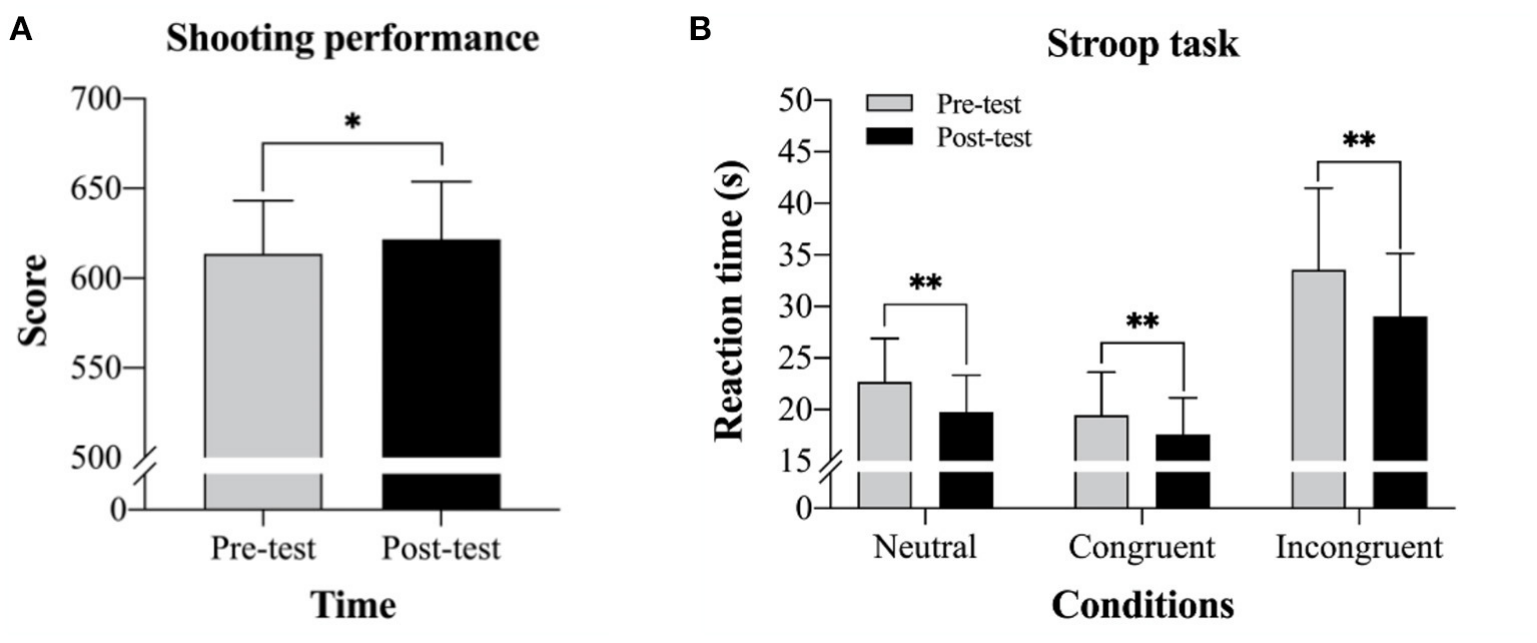
Sport and cognitive function performance results in the pre-test and the post-test following the mindfulness-based peak performance (MBPP) program. **(A)** Comparison of shooting score (*M* ± SE) results between the pre-test and post-test and **(B)** Comparison of Stroop task condition results between the pre-test and post-test. *p < 0.05, **p < 0.01.

The paired sample *t*-tests revealed a significant difference between the pre-test and post-test results [*t*(22) = −3.58, *p* = 0.002, *d* = 0.27], with higher shooting scores in the post-test (*M* = 621.70, *SD* = 32.08) compared to the pre-test (*M* = 613.48, *SD* = 29.72) (Figure 1A).

### Cognitive Function Performance: Stroop Task

One of 23 participants was unable to complete the Stroop task, which resulted in total sample of 22 participants for the final analysis. Regarding the neutral condition, the paired sample *t*-tests revealed a significant difference between the pre-test and post-test results [*t*(21) = 6.90, *p* < 0.001, *d* = 0.71), with a shorter mean reaction time for the post-test (*M* = 19.77, *SD* = 3.75) compared to the pre-test (*M* = 22.69, *SD* = 4.24). For the congruent condition, the paired sample *t*-tests also revealed a significant difference between the pre-test and post-test results [*t*(21) = 5.05, *p* < 0.000, *d* = 0.51], with a shorter mean reaction time for the post-test (*M* = 17.59, *SD* = 3.72) compared to the pre-test (*M* = 19.62, *SD* = 4.21). Finally, for the incongruent condition, the paired sample *t*-tests likewise revealed a significant difference between the pre-test and post-test results [*t*(21) = 4.56, *p* < 0.001, *d* = 0.64], with a shorter mean reaction time for the post-test (*M* = 29.01, *SD* = 6.45) compared to the pre-test (*M* = 33.70, *SD* = 8.05) (Figure 1B).

### Dispositional Mindfulness

**Figure 2:**
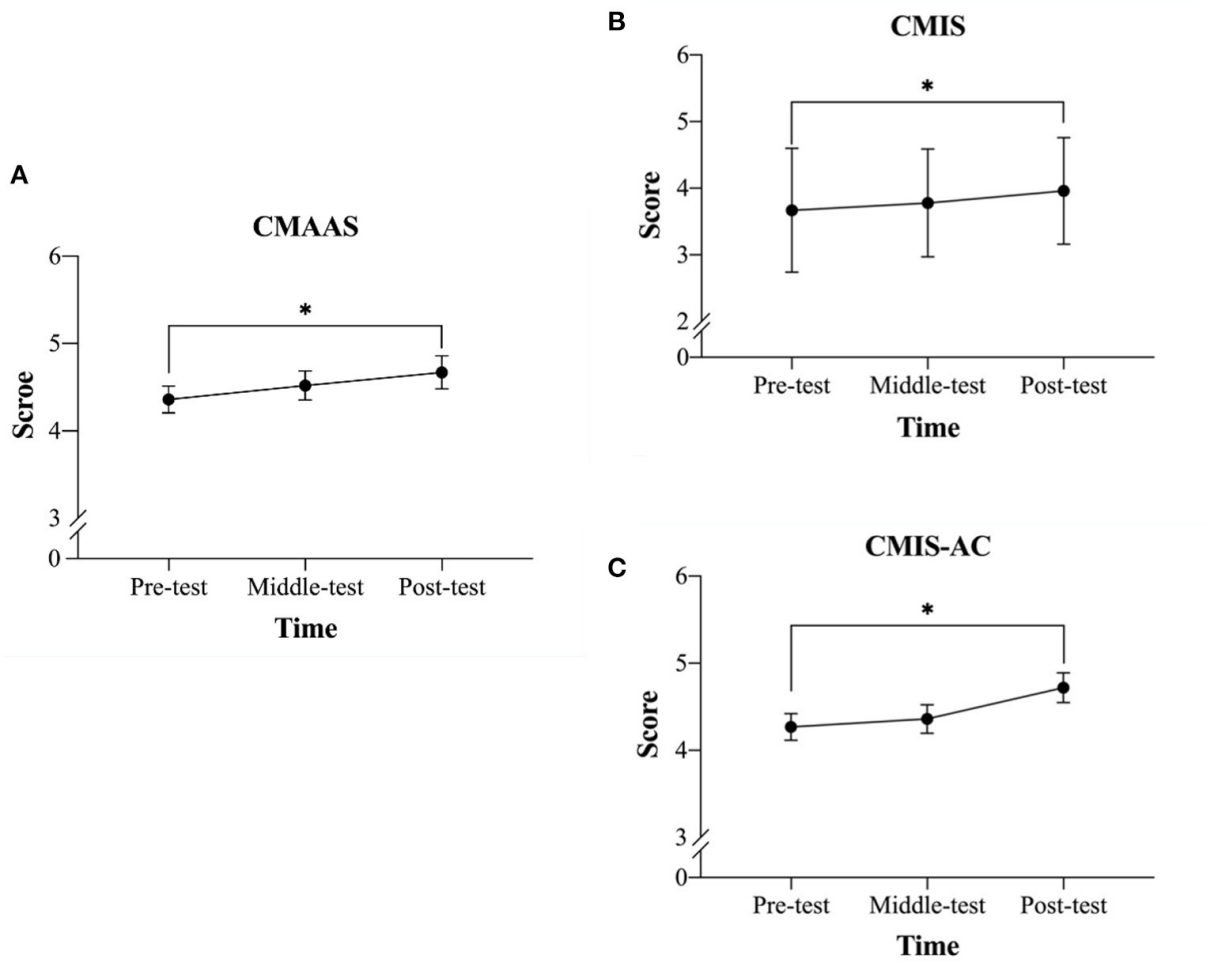
Changes in mindfulness levels across three time points before, at the mid-point of, and after the mindfulness-based peak performance (MBPP) program. **(A)** Comparison of the scores (*M* ± SE) on the Chinese version of the Mindful Attention Awareness Scale (CMAAS) across the three time points, **(B)** Comparison of the total scores on the Chinese version of the Mindfulness Inventory in Sport (CMIS) scale across the three time points, and **(C)** Comparison of the scores for the attentional control sub-dimension of the CMIS across the three time points. *p < 0.05.

A one-way ANOVA of the CMAAS revealed a significant difference across the three time points [*F*(2, 44) = 3.74, *p* = 0.032, *η*_*p*_^2^ = 0.15]. *Post-hoc* analysis Bonferroni adjustments revealed that the mean post-test score (*M* = 4.67, *SD* = 0.91) was significantly greater than the mean pre-test score (*M* = 4.36, *SD* = 0.74) (*p* < 0.05), while no other significant differences were observed (middle-test, *M* = 4.52, *SD* = 0.80) (Figure 2A).

A one-way ANOVA for the total score of the CMIS revealed a significant difference across the three time points [*F*_(2, 44)_ = 6.29, *p* = 0.004, *η*_*p*_^2^ = 0.22]. *Post-hoc* analysis Bonferroni adjustments revealed that the mean post-test score (*M* = 3.96, *SD* = 0.38) was significantly greater than the mean pre-test score (*M* = 3.67, *SD* = 0.44) (*p* < 0.05), while no other significant differences were observed (middle-test, *M* = 3.78, *SD* = 0.39) (Figure 2B).

A one-way ANOVA for the attentional control sub-dimension of the CMIS revealed a significant difference across the three time points [*F*_(2, 44)_ = 6.58, *p* = 0.003, *η*_*p*_^2^ = 0.23]. *Post-hoc* analysis Bonferroni adjustments revealed that the mean post-test score (*M* = 4.72, *SD* = 0.82) was significantly greater than the mean pre-test score (*M* = 4.27, *SD* = 0.74) (*p* < 0.05), while no other significant differences were observed (middle-test, *M* = 4.36, *SD* = 0.78) (Figure 2C). Meanwhile, a one-way ANOVA for the non-judgment sub-dimension revealed no significant differences across the three time points [*F*_(2, 44)_ = 6.22, *p* = 0.541, *η*_*p*_^2^ = 0.03; pre-test, *M* = 4.55, *SD* = 0.87; middle-test, *M* = 4.38, *SD* = 0.84; post-test, *M* = 4.56, *SD* = 0.89].

### Ruminations

**Figure 3:**
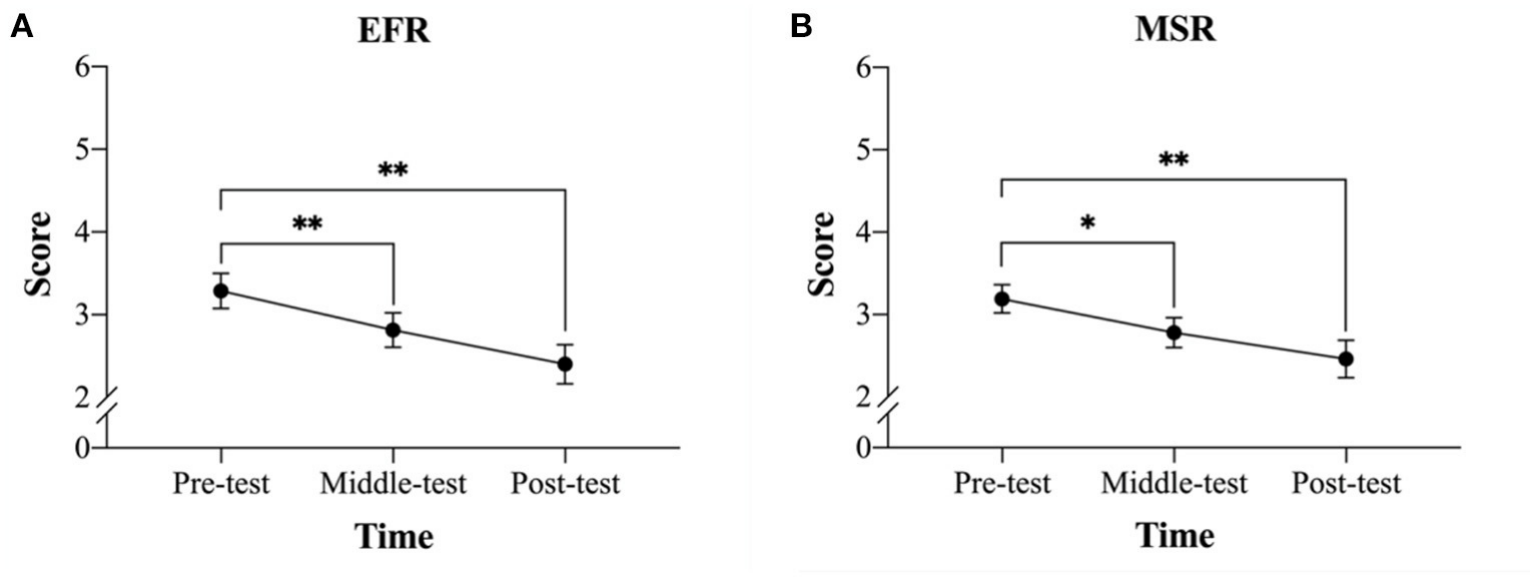
Changes in rumination score across three time points (*M* ± SE) before, at the mid-point of, and after the mindfulness-based peak performance (MBPP) program. **(A)** Comparison of the scores for the emotional-focused rumination (EFR) sub-dimension and **(B)** comparison of the scores for the meaning-searching rumination (MSR) sub-dimension. *p < 0.05, **p < 0.01.

A one-way ANOVA of the emotional-focused rumination sub-dimension revealed a significant difference across the three time points [*F*_(2, 44)_ = 16.46, *p* < 0.001, *η*_*p*_^2^ = 0.43]. *Post-hoc* analysis Bonferroni adjustments revealed that the mean pre-test score (*M* = 3.29, *SD* = 1.01) was significantly higher than the mean middle-test score (*M* = 2.81, *SD* = 1.00, *p* < 0.01) and the mean post-test score (*M* = 2.40, *SD* = 1.14, *p* < 0.001), while there was no significant difference between the middle-test and post-test means (*p* > 0.05) (Figure 3A).

A one-way ANOVA of the meaning-searching rumination sub-dimension revealed a significant difference across the three time points [*F*_(2, 44)_ = 16.46, *p* < 0.001, *η*_*p*_^2^ = 0.43). *Post-hoc* analysis Bonferroni adjustments revealed that the mean pre-test score (*M* = 3.19, *SD* = 0.82) was significantly higher than the mean middle-test score (*M* = 2.78, *SD* = 0.87, *p* < 0.05) and the mean post-test score (*M* = 2.46, *SD* = 1.09, *p* < 0.01), while there was no significant difference between the middle-test and post-test means (*p* > 0.05) (Figure 3B).

A one-way ANOVA of the instrumental rumination sub-dimension revealed no significant differences across the three time points [*F*_(2, 44)_ = 0.44, *p* = 0.650, *η*_*p*_^2^ = 0.02; pre-test, *M* = 4.04, *SD* = 0.63; middle-test, *M* = 4.00, *SD* = 0.75; post-test, *M* = 3.94, *SD* = 0.74].

### Correlation

The Pearson correlation analysis revealed a positive correlation between the shooting score and the total score of the CMIS (*r* = 0.426, *p* = 0.043), but the shooting score was not significantly correlated with the cognitive function performance or the other psychological outcomes.

## Discussion

The present study sought to determine the effects of an MBPP program on fine motor sport performance, to examine the potential role of psychological status, and to investigate the optimal dose of an MBI program for achieving desired changes in archery. The primary results revealed higher post-test shooting scores, cognitive function performances, and both general and athletic mindfulness levels compared to those for the pre-test. Additionally, decreased negative ruminations has revealed since the middle-test. Lastly, a positive correlation between the shooting scores and the mindfulness levels induced by the MBPP program was also observed.

### Shooting Performance

The finding of the present study that the MBPP program resulted in improvements in shooting performance was consistent with those of previous studies that utilized dart throwing (Zhang et al., 2016) and shooting (John et al., 2011), while also expanding upon studies that have indicated that MBIs can directly affect fine motor sports performance (Bühlmayer et al., 2017). Notably, the strength of this study was that performance in a simulated athletic competition was adopted as an objective assessment that provides a better understanding of the beneficial effects of MBI on performance, including better ecological validity (Gross et al., 2018; Josefsson et al., 2019). In a contrasting example, a previous study by Kaufman et al. (2009) failed to observe any effects of an MBI via the self-report approach in golfers and archers. Taken together with the results of our study, the results of that earlier study imply that the effects of MBIs can be more effectively evaluated with objective assessments that reflect the reality of athletic performance rather than with subjectively biased self-reports.

### Cognitive Functions

The archers who participated in the present study showed enhanced Stroop task performances across all the Stroop conditions (i.e., the neutral, congruent, and incongruent conditions) after the MBPP program, suggesting that multiple aspects of the athletes' cognitive functioning, from basic information processing to inhibitory control, were simultaneously enhanced by the MBPP program (Moore and Malinowski, 2009; Allen et al., 2012; Nien et al., 2020). These results are crucial because better basic information processing (e.g., processing speed, visual search ability) and EF have been found to distinguish elite athletes (Scharfen and Memmert, 2019) and elite gamers from low-ranked athletes and gamers and are hallmarks of peak performance (Toth et al., 2019). The improved inhibitory control induced by an MBI may be affected by reducing conflict monitoring in neural processes. For instance, Nien et al. (2020) reported that athletes who attended mindfulness training exhibited higher accuracy scores across all conditions of the Stroop task and smaller N2 amplitudes (a sensitive marker of response inhibition that reflects the conflict monitoring related to cognitive control) than those in a control group. Additionally, a neuroimaging study also found that, compared with relaxation training, an MBI increased the resting-state functional connectivity among EF-related brain networks (i.e., the dorsolateral prefrontal cortex and dorsal and ventral networks) in individuals with psychological distress (Taren et al., 2017).

Our results showed non-significant correlations between the investigated cognitive functions and shooting performance, which is inconsistent with previous studies (Verburgh et al., 2014; Huijgen et al., 2015; Sakamoto et al., 2018). It should be noted that the majority of those past reports were focused on gross motor sport performance (e.g., that of soccer athletes), whereas fine motor sport performance (i.e., archery performance) was focused on in this study. Jacobson and Matthaeus (2014) have suggested that different types of sport experiences may moderate the sport effects on cognitive performance. Moreover, the nature of the cognitive tests used may also result in differences. For example, several studies have employed the stop-signal task to detect motor inhibition (Verburgh et al., 2014; Huijgen et al., 2015); however, we utilized the Stroop task to measure selective attention-related inhibitory control. Further studies are thus suggested in order to better understand the relevance of the sport and cognitive assessments applied.

### Psychological Outcomes

The findings that the archers exhibited greater general as well as athletic mindfulness levels in the post-test compared to the pre-test not only support previous findings regarding the beneficial effects of MBIs in athletes (Bühlmayer et al., 2017), university students (Dawson et al., 2019), persons with psychosis (Jansen et al., 2020), and patients with cancer (Xunlin et al., 2020), but also demonstrate that comprehensive improvement in different types of mindfulness can be induced by MBIs. The results also imply that the MBPP program used is effective in increasing mindfulness levels in athletes. Furthermore, a positive correlation between athletic mindfulness, but not general mindfulness, and shooting performance, was demonstrated, suggesting that athletic mindfulness levels may be considered a potential mediator affecting fine motor sport performance, a finding which extends to past studies that employed basketball free throw performance (Gooding and Gardner, 2009) and middle-distance running performance (Jones and Parker, 2016). This result may implicate that athletic mindfulness is more suitable for assessing the sport performance of athletes, compared with general mindfulness (Josefsson et al., 2019). On the other hand, the results showed a significant increase in the mindfulness level, which means archers improved their ability to concentrate on their present-moment experiences, which may improve the archer's coping skills, relative to a variety of sport-related challenges from internal and external events (Josefsson et al., 2017).

Furthermore, the MBPP program reduced the negative rumination levels of the participating archers, a result that is consistent with those of previous studies using MBSR and mindfulness-based cognitive therapy programs (Campbell et al., 2012; Frostadottir and Dorjee, 2019). Decreased rumination has been linked to increases in mindful attention and enables individuals to focus their attention on present-moment environmental cues (Campbell et al., 2012). Reduced rumination has also been found to be associated with superior cognitive performance (De Lissnyder et al., 2010; Zhang et al., 2019), which may contribute to athletic performance enhancement in athletes. Indeed, decreases in negative ruminations are also highly relevant to coping skills for athletic performance, and reducing the manifesting of unwanted emotion (Josefsson et al., 2017). Notably, both emotional-focused and meaning-searching aspects of ruminations were improved following the MBPP program in this study, and given that these two dimensions of rumination may be related to the emotional regulation of stressors in competitions (Rood et al., 2009; Josefsson et al., 2017), our findings indicate that the MBPP program can improve upon poor adjustment and maladaptive coping in emotional regulation. Specifically, the athletes who have a relatively higher mindfulness level and less negative ruminative thoughts, and who may experience a lower intensity of negative emotions at the same time, by achieving such balanced mental states could perhaps be better enabled to focus completely on goal-directed behaviors, in order to achieve their optimal performance in the competitions (Thompson et al., 2011; Josefsson et al., 2017). Taken together, the results of this study indicate that a reduction of rumination can be induced by MBIs, providing additional evidence that athletes should be encouraged to engage in MBIs to prevent negative emotions and achieve optimal athletic performance.

### Dose-Response Relationship

One of the strengths of the present study was that it examined the dose-response relationship between an MBI and its associated changes of psychological status (i.e., mindfulness and ruminations), with assessments at three time points (i.e., the pre-test, middle-test, and post-test) having been employed. Both general and athletic mindfulness levels were improved at the post-test, meaning that the improvements of mindfulness have occurred in the late stage of the MBPP program. Nevertheless, this result does not correspond with findings of the previous clinical study by Roos et al. (2019), whereas there was found that mindfulness level was improved after the eighth session of MBPP in the present study, which is not supporting their results, that more than two sessions only could be associated with higher mindfulness level. The inconsistent findings might result from the differences in components of the programs and characteristics of their practitioners.

Contrarily, decreased ruminations were observed just after middle-test and maintained until the end of the MBPP program, suggesting that the rumination levels could have been improved only in the early stage of the MBPP program. This finding was found to be partly similar to results of Roos et al. (2019), in that they found that attending more than two sessions of mindfulness could predict the improvement of mental health. It should be noted that although a 4 weeks program was used in this study, the frequency of sessions was set at twice per week, for an overall dose that was similar to those of past studies (Creswell, 2017; Roos et al., 2019). These findings provide an initial explanation of the dose-response relationships of the MBPP program, in terms of training frequency and overall length, on different psychological outcomes in athletes.

Alterative dose-response relationships associated with session duration and frequency may be presented. In contrast to Kaufman et al. (2009) they observed no effect on performance following a mindfulness program constituted of 2.5–3 h sessions, done weekly, for 4 weeks (i.e., four times with longer session times), whereas we observed a significant improvement in archer performance following a twice per week 1 h session, for 4 weeks (i.e., eight times with a shorter time). The difference may be associated with session duration, in which studies have suggested that long mindfulness sessions may produce hindrances (e.g., self-critical or confusing reactions) during practice (Banerjee et al., 2017; Strohmaier et al., 2021). These findings suggest that mindfulness programs with short duration and more frequency may lead to a larger effect on performance, and this requires further investigation.

### Strength and Limitations

The strengths of the present study include the fact that its examination of the effects of the MBPP program on athletic performance had better ecological validity than some previous studies, as well as its investigation of many aspects of psychological status and the dose-response relationship of the MBPP program through multiple assessments. However, our results should be also interpreted cautiously for several reasons. First, the lack of a control group prevents the drawing of any cause-effect interpretations. Additionally, given the relatively small number and unbalance of gender of the elite level participants engaged in a specific sport, which may limit statistical power, and cause the generalizability of the findings to be limited. As such, in order to better understand how the MBPP program might affect the investigated outcomes, further studies using quasi-experimental designs or randomized controlled trials with larger sample sizes from more sport types are needed in the future. Another limitation is that the present study did not administer the structured post intervention follow-up, which may not adequately measure the effectiveness of the MBPP program across the entire training progress of participants, and its carry-over effects. Future research should employ a longitudinal design with a follow-up, in order to better understand the efficacy and effectiveness of the MBPP program for athletes. Third, despite the simulated competition having been adopted for the purpose of assessing the participants' performances objectively, the limited authenticity of the simulated archery competition may mean that the study results are not generalizable to any highly competitive genuine competitions. Lastly, despite that each session in the MBPP program included a discussion section, the data of running the program (e.g., interaction between the experts and the athletes) were not collected. It would be required to use qualitative methods so as to better and more deeply understand the applications of the MBPP program in future works.

## Conclusion

The present study provides empirical and preliminary evidence that supports the benefits of an MBPP program for archers in terms of shooting performance, multiple cognitive functions, and psychological outcomes. Additionally, the MBI ameliorated mindfulness and ruminations in the late-stage and middle-stage, respectively. Lastly, the MBPP program may be a promising approach for enhancing athletic performance, suggesting that athletes and coaches could integrated the MBPP program into sport training routines. However, further studies considering performance-relevant outcomes, and implemented with high-quality methodology, are needed in order to replicate the findings of the present study.

## Data Availability Statement

The original contributions presented in the study are included in the article/Supplementary Material, further inquiries can be directed to the corresponding authors.

## Ethics Statement

The studies involving human participants were reviewed and approved by Center for Research Ethics of National Taiwan Normal University. The patients/participants provided their written informed consent to participate in this study.

## Author Contributions

T-YW, Y-CC, H-CC, and Y-KC contributed to the conception of the work. T-YW, Y-CC, H-CC, and Y-KC contributed to the design of the work. J-TN, GK, C-HW, and H-CC conducted the literature search, selection, data extraction, and analysis. T-YW, J-TN, GK, and C-HW wrote the first draft of the manuscript with support from H-CC. All authors contributed to the manuscript revisions and agreed with final approval of the version.

## Conflict of Interest

The authors declare that the research was conducted in the absence of any commercial or financial relationships that could be construed as a potential conflict of interest.
