# Supplementary material for: The Effects of Mindfulness-Based Intervention on Shooting Performance and Cognitive Functions in Archers
Source: Front Psychol. 2021 Jun 24;12:661961. doi: 10.3389/fpsyg.2021.661961 (PMC8268485; doi:10.3389/fpsyg.2021.661961)
Supplement: Supplementary file 1 [file Data_Sheet_1.docx]

**Appendix**

**Summary Outline of the Mindfulness-Based Peak Performance Protocol**

1. **Session 1: Introduction explaining what mindfulness is**
   1. Motivation: brief story
   2. Introduction and psychoeducation regarding the mindfulness program
      1. Purpose of mindfulness-based peak performance (MBPP)
      2. What is mindfulness? (awareness, paying attention on purpose, being in the present moment, and experiencing things non-judgmentally)
      3. Benefits of mindfulness
   3. The practice of mindfulness
      1. Mindful check-in exercise
      2. Raisin exercise
      3. Group discussion
   4. Assigning homework
2. **Session 2: Mindfulness in sports contexts**
   1. Review of homework and brief discussion
   2. Motivation: brief story
   3. Keys to successful sport performance
      1. Factors affecting outcomes in competitions
      2. Choking in archery
      3. Effects of mindfulness on peak performance
   4. The practice of mindfulness
      1. Raisin exercise
      2. Mindful breathing exercise
      3. Group discussion
   5. Assigning homework
3. **Session 3: Let mindfulness be a habit**
   1. Review of homework and brief discussion
   2. Motivation: brief story
   3. How are habits formed?
      1. Steps in establishing habits
      2. Neuroplasticity
   4. The practice of mindfulness
4. Mindful breathing exercise
5. Body scan exercise
6. Group discussion
   1. Assigning homework
7. **Session 4: Cope with stress**
   1. Review of homework and brief discussion
   2. Motivation: brief story
   3. How does stress affect sports performance?
      1. What is stress?
      2. Stress reactions
      3. Effects of mindfulness on stress
   4. The practice of mindfulness
      1. Body scan exercise
      2. Seated meditation exercise
      3. Group discussion
   5. Discussion of homework
8. **Session 5: True rest**
   1. Review of homework and brief discussion
   2. Motivation: brief story
   3. Importance of rest
      1. How does a lack of rest affect sports performance?
      2. The brain’s dark energy: The default mode network (DMN)
      3. Effects of mindfulness on the DMN
   4. The practice of mindfulness
      1. Mindful breathing exercise
      2. Mindful listening exercise
      3. Group discussion
   5. Assigning homework.
9. **Session 6: Pay attention in the present moment**
   1. Review of homework and brief discussion
   2. Motivation: brief story
   3. Importance of attention in sports contexts
      1. Types of attention
      2. Attention bias
      3. Effects of mindfulness on attention
   4. The practice of mindfulness
      1. Seated meditation exercise
      2. Mindful listening exercise
      3. Group discussion
   5. Assigning homework.
10. **Session 7: Emotional regulation**
    1. Review of homework and brief discussion
    2. Motivation: brief story
    3. Emotions in sports contexts
       1. What is are emotions?
       2. Attention bias
       3. Effect of mindfulness on attention
    4. The practice of mindfulness
       1. Mindful listening exercise
       2. Mindful walking exercise
       3. Group discussion
    5. Assigning homework
11. **Session 8: Successful decision making**
    1. Review of homework and brief discussion
    2. Motivation: brief story
    3. Importance of executive function in sports contexts
       1. What is executive function?
       2. Executive function and peak performance
       3. Effects of mindfulness on executive function
    4. The practice of mindfulness
       1. Mindful walking exercise
       2. Mindful Bagua Dǎo yǐn exercise
       3. Group discussion
    5. Summary
       1. What are the benefits of mindfulness for you?
       2. Discussion of strategies for continued practice
