# Supplementary material for: The Effects of Mindfulness-Based Intervention on Shooting Performance and Cognitive Functions in Archers
Source: Front Psychol. 2021 Jun 24;12:661961. doi: 10.3389/fpsyg.2021.661961 (PMC8268485; doi:10.3389/fpsyg.2021.661961)

Appendix II
Short notes for Raisin exercise and Mindful Bagua Dǎo yǐn exercise

- <u>Raisin Exercise</u>
  - In the raisin exercise, participants listened to a shortened Chinese version (approximately 5 min) of Jon Kabat-Zinn's (2006) mindful raisin-eating exercise, which is a common and classical mindfulness exercise used in clinical and non-clinical settings.
  - The clients would be invited to voluntarily aware of the raisin through all senses, and what you can see (e.g., color or texture), touch (soft or hard), smell (e.g., scents or fragrances), and taste (sour or sweet) in the present moment. This raisin exercise encourages present-moment awareness of the experiences as a way to step out of the habitual unconsciousness of behavior.

- <u>Mindful Bagua Dǎo yǐn exercise</u>
  - Mindful Bagua Dǎo yǐn exercise is a relatively new form of mindfulness exercise that is developed based on the Bagua Zaang (i.e., one of the Chinese Internal Martial Arts) (https://baguadaoyin.wixsite.com/baguadaoyin/class).
  - Specifically, Mindful Bagua Dǎo yǐn exercise is one of the foundational exercises in Bagua Zaang, that aims to loosen up the joints in order to increase flexibility and mobility while holding static postures to be mindful. During the exercise, Bagua Dǎo yǐn typically contains holding fixed postures and slow movements to create body experiences or senses (e.g., horse stance and bow-arrow stance), and stretch the whole body and limbs to create fascial twists and relax. Importantly, clients would be instructed that placed on bringing full awareness to the moment-to-moment experience and be the mindful attitude towards the body.

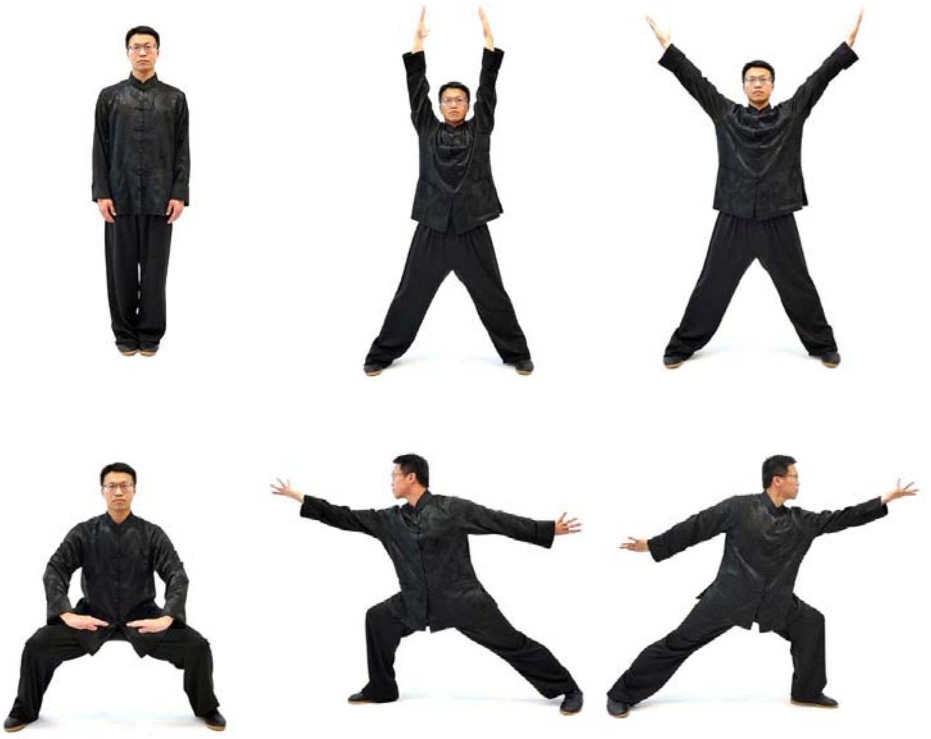

Supplement: Supplementary file 2 [file Data_Sheet_2.pdf]
